# Integrating community health workers into HIV care clinics: a qualitative study with health system leaders and clinicians in the Southern United States

**DOI:** 10.1186/s12913-024-11381-6

**Published:** 2024-11-01

**Authors:** Shannon M. Fuller, Emily A. Arnold, Jessica Xavier, Chidinma A. Ibe, Wayne T. Steward, Janet J. Myers, Greg Rebchook, Kimberly A. Koester

**Affiliations:** 1 Department of Medicine, Division of Prevention Sciences, University of California, San Francisco, San Francisco, CA USA; 2 Department of Health, Behavior and Society, Johns Hopkins Bloomberg School of Public Health, Baltimore, MD USA; 3 Department of Medicine, Division of General Internal Medicine, Johns Hopkins University School of Medicine, Baltimore, MD USA

**Keywords:** Community health workers, HIV, Care integration, Qualitative research

## Abstract

**Background:**

Community health workers (CHWs) can support patient engagement in care for a variety of health conditions, including HIV. This paper reports on the experiences of HIV clinics and health departments that integrated CHWs into their health systems as part of a capacity-building initiative to address HIV-related disparities in the United States.

**Methods:**

Semi-structured interviews were conducted with key informants (*n* = 14) in two Ryan White HIV/AIDS program jurisdictions: Mississippi (jurisdiction covers the entire state) and the city of New Orleans, Louisiana. This work was part of a larger evaluation of an initiative that used a Learning Collaborative model to facilitate the implementation of evidence-informed interventions to address HIV care continuum gaps in four jurisdictions. The two jurisdictions that focused on integrating CHWs into HIV care clinics and support service agencies were selected for this sub-analysis. Interview participants included HIV clinic leaders and staff, health department leaders, and other Learning Collaborative leaders. Interview transcripts were coded and analyzed for themes related to the acceptability, feasibility, and perceived impact of CHW integration.

**Results:**

Overall, participants expressed interest in having support from CHWs at HIV clinics and service agencies to assist with patient retention and engagement efforts. However, there were challenges integrating CHWs into existing systems (e.g., gaining access to electronic health records, changing policies to conduct home visits, and clarifying roles and scope of work). Negotiating contracts and accessing funding for CHW positions presented major challenges that often contributed to turnover and conflicts around scope of practice. When health departments leveraged existing funding streams to support CHW positions, the clinics and agencies where the CHWs worked had limited flexibility over the hiring process.

**Conclusions:**

Our findings reinforce the value and acceptability of CHWs as part of the workforce in HIV clinical and support service settings; however, integrating CHWs into clinics and service agencies required effort. Training the CHWs was not sufficient; other staff and clinicians had to understand the role of CHWs to facilitate their integration into health systems. Resources are needed to support organizations in incorporating CHWs effectively, and long-term, flexible sources of funding are necessary for these positions.

**Supplementary Information:**

The online version contains supplementary material available at 10.1186/s12913-024-11381-6.

## Background

Community health workers (CHWs) are frontline public health workers who possess a deep understanding of and trust within the communities they serve, allowing them to bridge gaps between medical and community settings to enhance access to quality care [1]. Although the National Association of Community Health Workers and the Community Health Worker Core Consensus (C3) Project have characterized the core competencies and scope of practice for CHWs in effort to standardize the terminology ascribed to the workforce in the US [2], there continues to be considerable variation in how CHW positions are described. At the time this study was conducted, the Centers for Disease Control and Prevention (CDC) listed 22 different job titles under the umbrella of “community health worker” in its training module [3]. CHWs may be referred to as outreach workers, health promoters, and peer navigators. Positions can be on a paid or volunteer basis and can vary widely in scope (e.g., focused on one disease or condition, or focused on health promotion more generally). Despite this variation, what CHWs have in common is that they often share lived experiences or other attributes with the communities they support or otherwise have a uniquely close understanding of those communities. This nuanced understanding, coupled with their interpersonal skills, allows them to help clients navigate multi-level barriers to addressing medical and social needs.

CHW-led interventions are frequently cited as effective solutions to address healthcare workforce shortages and are uniquely positioned to connect structurally disenfranchised populations to health education and services [4, 5]. Studies have shown CHWs to be effective in reducing health disparities across a variety of conditions and contexts [5, 6]. A 2010 Cochrane Review found that CHWs have been successful in increasing uptake of vaccinations, promoting breastfeeding, and supporting self-management of chronic conditions [6]. More recently, a large, multi-site evaluation in the US found that after integrating CHWs into HIV primary care settings, visit attendance increased significantly from approximately 50–85% of participants having at least one visit, and viral suppression increased significantly from 22 to 44% of study participants [7].

CHWs have long operated in community settings both globally and in the US, though their integration into clinics and care teams is relatively new [8]. The 2010 Patient Protection and Affordable Care Act (ACA), which most notably expanded access to health insurance, also included provisions for better coordination of care – specifically by including CHWs in interdisciplinary health care teams – to reduce health care spending and costly hospital admissions [9]. While integration can be broadly defined, full integration includes having CHWs located at the clinic, designated as part of care teams, and having shared access to electronic health records [10]. There are several benefits of incorporating CHWs into clinics and health systems. Integration can also help CHWs be more effective in supporting patients, as it can facilitate better ability to assist in access to resources available through health systems [11, 12]. When done well, integration may also improve the experiences of CHWs by providing opportunities for job security and professional growth [13]. General best practices for incorporating CHWs into clinical settings have been documented, including clearly defining roles among clinic staff, providing supportive supervision, and ensuring ongoing training for CHWs [11, 13–17]; however, less is known about the processes for *how* to develop and sustain these models, and how health system leaders and other clinicians experience CHW integration.

This paper reports on experiences from two Health Resources and Services Administration (HRSA) Ryan White HIV/AIDS Program (RWHAP)-funded jurisdictions in the US that focused on integrating CHWs into HIV clinics to improve retention and engagement in care. There is limited research on the integration of CHWs into HIV clinics. In the US, most of the research to date has come from a three-year initiative funded by the Health Resources and Services Administration (HRSA) in 2016 to provide training and support to integrate CHWs into care teams at 10 RWHAP-funded clinics across the country. That initiative laid important groundwork on the effectiveness and value of CHWs as part of HIV care teams, and implementation strategies for their integration into HIV care settings [7, 14, 18].

The current study was funded by a more recent HRSA initiative (2019–2023): the “Capacity Building in the Ryan White HIV/AIDS Program to Support Innovative Program Model Replication,” geared toward enhancing the capacity of RWHAP recipients and subrecipients to replicate evidence-informed interventions [19]. Jurisdictions represented a sample of priority regions identified under the Ending the Epidemic (EHE) Initiative due to increasing transmission of HIV and growing disparities in key populations [20]. The four jurisdictions were Atlanta, Georgia; the state of Mississippi; Las Vegas, Nevada; and New Orleans, Louisiana. In partnership with the University of California San Francisco (UCSF), jurisdictions used a Learning Collaborative model [21] to choose and implement evidence-based interventions. Two of the jurisdictions, Mississippi and New Orleans, chose to focus on employing community health workers (CHWs) to address disparities in HIV care retention. Unlike the CHW initiative described earlier, funding was not directed to implementing and evaluating a specific intervention. Rather, funding was provided to build system-level capacity to promote broader implementation of care models already determined to be effective in other settings (ideally, in other RWHAP sites across the country). Funding was used to convene stakeholders across the jurisdictions in Learning Collaboratives and coordinate existing resources, such as previously developed intervention manuals and other federal and local funding streams to support implementation. This structure meant that interventions were not confined by strict parameters, and there was variation in how programs operated across the different jurisdictions. Learning Collaboratives included “Learning Sessions” that convened jurisdiction leaders and representatives from participating clinics and agencies every few months as well as one-on-one or small group technical assistance delivered or organized by Learning Collaborative leadership to participating sites as needed throughout from the project. Another important distinction was that planning occurred primarily at the jurisdiction level. Although clinics and service agencies were engaged through the Learning Collaboratives’ Planning Body meetings and Learning Sessions, the integration of CHWs was mostly a top-down endeavor led by state and local health departments and other leaders of the jurisdiction. In one jurisdiction, the CHWs were hired by the state under a different initiative and then assigned to the clinics that were part of the Learning Collaborative. In the other jurisdiction, clinics hired CHWs directly based on referrals from the Learning Collaborative leadership (including representatives from the health department and statewide CHW association), who led efforts to train a new cadre of CHWs focused on HIV care and prevention.

As part of a larger implementation science evaluation of the HRSA capacity building initiative, we conducted a qualitative sub-study to explore the motivations underlying the decisions to implement a retention intervention using CHWs, the barriers and facilitators to incorporating CHWs into existing HIV services as well as identifying best practices for optimizing the integration of CHWs into HIV care teams and health systems. As the CHW workforce continues to grow, findings from this study can help to inform the coordination and management of efforts to integrate CHWs into HIV care and related services at state and local levels.

## Methods

### Sample

We conducted qualitative interviews across all four of the jurisdictions as part of our multi-method evaluation study. For this paper, we draw from interviews and observations conducted in the two RWHAP jurisdictions – New Orleans and Mississippi – that focused on CHWs. Within each jurisdiction, we used purposive sampling to recruit and interview key informants who included health department leadership and staff, other Learning Collaborative Planning Body members, and staff and leaders from participating clinics/agencies. Interviews were conducted between September 2021 and March 2022, approximately 1.5 to 2 years after the projects launched.

### Data collection

Our study procedures were reviewed by the UCSF institutional review board and received a “not engaged in human subjects research” determination because our work was focused on quality improvement. Nonetheless, we followed standard research procedures in recruitment, interviewing, and data protection. Interview participants were recruited via email by a member of the qualitative evaluation team. All participants provided verbal informed consent to participate and were offered a $75 gift card to participate.

Interviews were conducted by a team of experienced qualitative researchers (KK, EA, JX, SF, AM) using semi-structured interview guides developed by the research team. Semi-structured interview guides were created to capture constructs from Proctor’s framework of implementation outcomes [22] that guided our larger project’s overall evaluation design. The same general guide (available as a Supplemental file) was used across all jurisdictions and participants, with some adjustments made depending on the individual’s role or location. Following Proctor’s framework, we inquired about perceived outcomes related to implementation (appropriateness, acceptability, adoption, feasibility, and sustainability). We also explored the perceived impact of the intervention(s) and the Learning Collaborative. Interviews took place via Zoom and lasted approximately 45–75 min.

Qualitative researchers also conducted observations of the Learning Sessions conducted across each jurisdiction and recorded field notes. Learning Sessions were one-to-two-day meetings, mostly held over Zoom due to the COVID-19 pandemic, that convened jurisdiction-level leadership and representatives from participating clinics and agencies in the Learning Collaborative. Each jurisdiction held a Learning Session every 3–6 months. The qualitative observers and their roles were introduced at each Learning Session. Observations provided a way to gain understanding of the interpersonal relationships and contextual information about the participating sites to assist with data interpretation. Observations also helped to identify potential key informants and follow-up questions for the interviews. Because the interviews were only conducted at one point, the observations also provided insight into the ongoing implementation of the projects develop during the final months of the initiative.

### Analysis

We conducted a thematic analysis [23]. First, interviews were audio-recorded and transcribed verbatim. Authors SF, KK, EA, and JX developed a codebook that applied across all the jurisdictions for the larger initiative. The codebook consisted primarily of a priori codes based on Proctor’s framework for implementation outcomes [22] as well as the interview guide, which focused on understanding stakeholder experiences in the capacity-building initiative. We also included codes based on themes that emerged during the data collection process. Authors SF and KK coded all transcripts in Dedoose [24], a cloud-based application used for data analysis to facilitate data management and extraction of coded excerpts. The research question for this sub-study emerged from the coding process and the lead author’s interest in studying CHW programs. For this study, all excerpts under the following codes were reviewed and summarized: intervention narrative (a broad code that captured all narratives about the intervention(s) implemented in the jurisdiction and covered perceptions of intervention appropriateness and fit), implementation barriers, implementation facilitators, acceptability, adoption, feasibility, and sustainability. Looking across all the summaries, the lead author prepared an analytic memo that combined information across the key codes. The analysis focused on identifying common themes related to implementation experiences and lessons learned. These themes were subsequently cross-mapped to Proctor implementation outcomes (appropriateness, acceptability, adoption, feasibility, and sustainability) which are defined in Table 1 of the results section. Findings were discussed with the qualitative team, including those who had engaged in Learning Session observations to ensure comprehensive analysis and interpretation of the data. We conducted a member check to review findings with site coaches and a subset of participants to ensure that our results appropriately characterized their experiences.

## Results

### Sample overview

Our sample included a total of 14 key informants (5 participants from Mississippi and 9 from New Orleans). Participants were involved in leading the Learning Collaborative projects at the jurisdiction level (*n* = 6) or representing a clinic/agency that was participating in the collaborative (*n* = 8). Given the limited number of individuals working in different roles, state identifiers have been removed from examples and descriptions below to protect confidentiality.

### Program descriptions

The program structure and scope of the CHW role varied widely both across and within the two jurisdictions. For example, one jurisdiction provided training so that outreach workers, navigators, and case managers working in the HIV care and prevention field could receive training in CHW competencies. The training was guided by the Community Health Worker Core Consensus (C3) Project’s recommendations, which aim to standardize the core qualities, skills, and roles of CHWs [25]. The jurisdiction also provided training in the basics of HIV care and prevention for trainees who were new to the HIV field. The other jurisdiction used enhanced federal funding to create new CHW positions hired through the jurisdiction’s health department. CHWs were then placed in clinics to work with and support that facility’s providers and clients. These new CHW positions were intended to improve reliable client engagement of patients in care and prevention. Both jurisdictions shared a common interest in leveraging existing resources to promote sustainability. The Learning Collaborative served as a forum to assist participating clinics and agencies with implementation. Technical assistance and capacity building from UCSF was directed to jurisdiction leaders, who were then responsible for managing and overseeing site-specific training and other support.

Key informants described the breadth of tasks that CHWs managed, including finding patients who were out of care, linking patients to services for medical and social needs, providing emotional support, helping patients navigate complex care systems, understanding and addressing barriers to care, and guiding care teams in understanding the patient’s lived experience. CHWs were frequently characterized as critical to helping clinical teams develop and adjust patients’ care plans because of their nuanced understanding of each patient’s unique resources and needs. CHWs often gleaned insights into contextual factors that influenced engagement in care through home visits. Other roles of the CHWs included advocating for patients, conducting testing and outreach, helping them prepare for clinic appointments, and providing education related to HIV and general health.

### Implementation themes: overview

The following sections highlight the major themes related to participants’ experiences leading or implementing CHW integration projects. Table 1 shows the cross-mapping of these themes to implementation outcomes from Proctor’s framework. First, narratives around the motivations for the project at both the jurisdictional and clinic levels touch on perceptions of the *appropriateness* of the intervention as well as its ultimate *adoption* across the different locations. Second, training for CHWs and other providers was connected to the *acceptability* of integration. Third, role clarification was related to both the *acceptability* and perceived *feasibility* of the intervention, and it was often a more difficult process than clinic leaders anticipated. Fourth, discussions of funding, hiring, and CHW retention challenges illuminated perceptions around the *feasibility* and *sustainability* of integration. Each of these connections is explained further in the sections below.

**Table 1:** Major themes and connections to implementation outcomes

Themes	Implementation Outcomes
1) Motivations for integrating CHWs into HIV clinics and service agencies	• Appropriateness • Adoption
2) Importance of training for CHWs and other providers	• Acceptability
3) Role clarification	• Acceptability • Feasibility
4) Funding, hiring, and retention challenges	• Feasibility • Sustainability
*Definitions (Proctor)*: **Appropriateness**: perceived fit or relevance of the intervention in a particular setting or for a particular target audience or issue **Adoption**: the intention, initial decision, or action to try to employ a new intervention **Acceptability**: perception among stakeholders that an intervention is agreeable **Feasibility**: extent to which an intervention can be carried out in a particular setting or organization **Sustainability**: extent to which an intervention is maintained or institutionalized in a given setting	

### Theme 1) motivations for integrating CHWs into HIV clinics and service agencies

During our interviews, we explored the reasons why jurisdictions chose to implement the interventions that they did. Participant narratives revealed perceptions of the *appropriateness* and the *adoption* of the intervention at the jurisdictional and clinic levels. Across the larger initiative, jurisdiction leaders had the option to implement any evidence-informed intervention or combination of interventions to address local gaps in access to HIV care. The state or local health departments participated in an intervention selection process through discussions with the UCSF capacity-building assistance team and local Learning Collaborative members including leaders in the HIV community, providers, and other professional staff. Some already had specific interventions in mind and used the initiative to facilitate uptake across clinics and agencies within their jurisdiction. Common reasons behind choosing to integrate CHWs were to expand the reach into the community and engage or re-engage patients, and to strengthen an existing workforce. The two jurisdictions represented in this analysis noted how they had access to a CHW workforce – many of whom had historically been funded through other initiatives – and saw an opportunity to further integrate these workers into their HIV care teams to improve their capacity to promote patient retention and engagement in care. At the jurisdictional leadership level, there was a high degree of perceived *appropriateness* and strong fit between the intervention goals and the needs and resources of the local region.

Once an intervention or combination of interventions was selected, community engagement meetings were held with local clinics and agencies, who could then opt-in to participate in the Learning Collaborative and implement the intervention. Importantly, there were varying degrees to which clinics and agencies felt they could participate in integrating CHWs into their teams. This meant that attitudes and decisions around intervention *adoption* were mixed. In some cases, participation was perceived as an obligation because it was heavily encouraged by jurisdiction leadership, and it could be used to meet a clinic’s quality improvement objectives. In another example, integration was explicitly written into CHW contracts, effectively mandating the participation of the clinic or agency. In either case, participation was often regarded as an obligation, which partially explains the initial mixed responses to intervention adoption.

Some clinics were immediately on board with integrating CHWs into their teams. For example, the executive director of a clinic described how they were motivated to try any new ideas that could address the HIV epidemic. They knew that CHWs had been effective elsewhere but had not been part of the workforce in the region or clinic. Their clinic found the CHW’s support to be immensely beneficial in expanding reach into the community and attributed the increase in patients to outreach attempts from the CHW. In the quote below, the executive director described the motivation to try something new and expand reach into the community.

*“I don’t see how we can achieve better results with the epidemic*,* a 40-year epidemic*,* if we don’t do things that we haven’t done before. Because especially in the South*,* we’ve not lacked for effort and passion. But we don’t get the results. So*,* why don’t we do something that we haven’t tried? … If we don’t get in the community and communicate to everyday folk*,* regular folk*,* our neighbors*,* what we’re doing*,* they’re not going to take advantage of any of the resources we’re out here fighting to provide. To me*,* community health workers’ outreach*,* that’s the key piece to actually bringing the community onboard and making sure that they’re aware that we’re here*,* aware of what we’re providing. Otherwise*,* we’re just a logo.”* – Clinic leader, executive director

Other key informants described workforce development as a major motivating factor for training and integrating CHWs. They saw this initiative as an opportunity to build clinic workforce capacity and support further training and development of CHWs in their area. One participant noted how the initiative provided an opportunity to expand CHW training into HIV specifically, and how that could have an added benefit in reducing HIV-related stigma in the broader community.

*“In [our state]*,* community health workers traditionally were only used in hypertension and diabetes. Now we have a complete community health workforce that’s focused primarily on HIV*,* too. . we’ve diversified the labor class and you have people who are of the community*,* look like the community*,* who can speak the language. … they can use the information they know when they have their church outreach programs*,* they can use the information on the weekends or when they’re hearing people dispel myths and misconceptions about HIV*,* they have the knowledge and the fortitude to say*,* hey*,* what you’re saying isn’t right. This is actually how that is. So taking the opportunity to have teachable moments. Because now we have an educated group of people about a particular issue in a community who can help to dispel some of those myths and address the stigma.”* – Project leader, jurisdiction level

Leaders who had previous experience as CHWs or had worked closely with CHWs were highly motivated to elevate the awareness of the CHW role in the health system. They had witnessed or personally experienced the challenges that CHWs faced, including not being taken seriously because of their non-medical training, and navigating tensions with other care team members about potentially overlapping roles. These experiences shaped the ways that they developed and ran their Learning Collaboratives. Some of the ways they drew from these experiences included building a statewide CHW Association to provide legitimacy and external support for CHWs, and by providing one-on-one coaching to clinical sites to advocate for CHWs and facilitate training and integration.

### Theme 2) importance of training for CHWs and other providers

Informants across both jurisdictions talked about prior experiences when CHWs were brought onto projects but not provided appropriate training. In these past projects, there was a blurring of roles across members of the care team that created confusion and sometimes resentment. By contrast, informants spoke about how the training and HIV 101 certification provided through the current initiative helped to ensure that all the CHWs were on the same page. An informant touches on these points below and notes how the Learning Collaborative in conjunction with a newly established CHW association helped support the CHWs through opportunities for peer-to-peer networking.

*“A number of years ago*,* [our region] did utilize community health workers*,* but there was not a training program. People who were CHWs at the time often ended up taking—how can I put it? Sometimes they acted as the provider with giving information*,* advice*,* recommendations. That was clear that they lacked sufficient training. What ended up happening in some cases*,* CHWs were resented because it was felt that they had a certain amount of power or autonomy. But it wasn’t the fault of the CHWs - I would say the state or the organization. [The CHW programs] were so new that you really didn’t know what to give them*,* what parameters to set. Some organizations were happy with the CHW taking on so much responsibility*,* while others felt kind of threatened by it … It would be more challenging if we didn’t have this community health worker model and this community health worker network [including the state CHW association] and a [HIV-specific] certification and pushing the training and making sure all the CHWs are on the same page. They have their own networks so they can communicate with peers*,* which didn’t exist before.”* – Leader at health department

Although the CHWs received extensive training to support their integration into HIV clinics, other members of the care team were not always properly trained or aware of how to collaborate with the CHWs. In the quote below, a clinic leader explained how they felt that the CHW was “kind of dumped on us” and that they did not know how to effectively incorporate a CHW into their clinic.

*“My biggest recommendation*,* and again*,* I don’t know if this wasn’t done. But I felt like*,* and maybe others felt like we had the CHW kind of dumped on us. A lot of us don’t have a lot of experience with how to really use a CHW. … If we’re going to focus on CHWs*,* then we really need a lot of training on how to successfully set up a CHW program because a lot of us don’t have it. Or we have people that act*,* functionally*,* as a CHW. But if you have all these case managers and medical case managers and eligibility people in the mix*,* the patients just get confused. And then the agency has a hard time setting it up. … I know us*,* as an agency*,* and I won’t speak for the other agencies*,* obviously. But I don’t think we were ready for a CHW. I feel like*,* actually*,* our system is fine. We were able to do a lot of good without it. Ended up being a good thing*,* but you know*,* I would love to have a better idea of how to implement them.”* – Clinic leader, chief medical officer

Another agency expressed some initial concerns about bringing on a CHW, as they were managing other competing priorities. They were also concerned about the hiring process, as the clinic did not have a choice in who was selected for the role due to the top-down nature of the CHW funding. There were concerns about trusting this new person with their patients and staff. Fortunately, in this case, it worked out and the participant expressed that the CHW had been a tremendous asset.

*“I have no problems telling you that we were leery about [bringing on the CHW]*,* it was concerning because of everything else that we were dealing with*,* because we were doing the new EMR and COVID and everything; bringing someone in that we didn’t have a say so in who they picked or what this background was and trusting them to come in and be around clients who are very sensitive and kind of embed themselves*,* but she has been a rock star. We just love her to pieces. She’s the sunshine to my day sometimes*,* you know?”* – Clinic leader, registered nurse and quality manager

The examples in this section show how the *acceptability* of CHW integration could change over time. Furthermore, acceptability was often tied to perceptions of how sufficiently clinics were provided training and technical assistance from the Learning Collaborative to support CHW integration into teams and clinic workflows.

### Theme 3) role clarification

Learning Collaborative leaders knew that role clarification was essential to the success of their CHW integration efforts and provided resources and technical assistance to support clinics with this process. However, role clarification was often a more extensive process than clinic informants expected, and more challenging than Learning Collaborative leaders had anticipated. The challenges described in this section highlight additional aspects of *acceptability* as well as perceptions of the *feasibility* of CHW integration in the context of HIV care. Effectively clarifying roles involved more effort than mapping client workflows and conducting simple introductions. One of the most successful examples of role clarification happened when clinic leadership provided clear direction to the care teams to include the CHW and created opportunities for the team members to work together, particularly when interacting with clients.

*“The community health worker was something totally new for us. So*,* one of our directors gave him full training [meaning a comprehensive orientation to the organization]. Also*,* she charged us to also work with him. He also had to meet with the housing manager*,* the case manager*,* the psychosocial counselor*,* the medical case manager*,* and a substance abuse counselor. So much so*,* we would travel together on Saturday*,* when we went out into the field to actually work with the clients. Because we’ve already built rapport with the clients*,* but they didn’t know who this new person was.”* – Counselor at a social services organization

The ability to provide services outside of a clinic setting is often seen as a distinct feature of the work of CHWs. However, there was a tension that some key informants expressed in the context of HIV care. When asked about implementation barriers, one participant described how their organization did not allow the CHWs to go into the community – they had to be exclusively based at the clinics. In fact, the idea of a CHW approaching a patient who was out of care at home was deemed nonnormative and problematic at this one site.

*“I don’t even know if we would send them out into the community even if they were allowed…. [Interviewer: Oh*,* interesting. Say more about that – what gives you pause about sending them into the community? ] … So*,* if that person is out of care maybe because they went somewhere else or whatever*,* but they’re still trying to remain status anonymous*,* we don’t want to send somebody out into the field to knock on their door on behalf of [our clinic] because that’s not something that your normal doctor would do. Like*,* my primary care provider is not coming knocking on my door because I haven’t seen him in a while.”* – Clinic leader, director of HIV outreach services

This concern may be unique for settings that offer HIV care exclusively and in regions where stigma poses a significant challenge. Settings that offered prevention or other services in addition to HIV care did not express this concern. Those sites felt that the ability of the CHW to deliver services in the community was a crucial way of expanding the reach of services (including HIV testing) and re-engaging people in care.

Overall, one of the major barriers to integrating CHWs was the lack of a clearly defined role, as exemplified by this disagreement around the extent to which they should be working in the community rather than in the clinic. The Learning Collaborative provided some structure to support role clarification. By convening participating clinics and agencies every few months in Learning Sessions, those organizations could learn from each other and comparatively see the benefits of working with CHWs. Additionally, the Learning Collaborative leaders in each state provided technical assistance and training to participating agencies as needed. In the quote below, a member of the Learning Collaborative leadership team described how they had to coach some clinical sites on the new vision for the CHW model.

*“[Some organizations that had worked with CHWs previously] had one idea of what the community health workers would do*,* and it was like a junior case manager. We explained*,* no*,* if they’re sitting down in your office*,* then they’re not doing what we’re hiring them for. If they’re just dealing with data or eligibility*,* that’s not what we hired them for. Let your case managers do that. These people have hands-on boots on the ground working with the clients.”*– Project leader, jurisdiction-level

Both jurisdictions mentioned the challenges of undoing perceptions of CHWs from prior projects. The examples above show the clarifications that needed to happen within clinics as well as from the health department to the clinics.

Even when roles were well clarified, it took time to build the systems so that CHWs could operate under the full scope of their intended role. For example, several rounds of approvals were often needed so that the CHW could perform scheduling and other tasks in the electronic medical record. There was often a bit of gatekeeping and lack of trust initially – CHWs were initially perceived as outsiders and their roles needed to be justified, particularly in terms of access to the electronic health record. When asked about challenges with the project, an informant located at a large organization explained that even though their administration was highly supportive of the CHW, it still took considerable time for the approvals to get set up and for trust to be established.

*“The only thing we had a little bit of time was getting approvals because it goes through 8 million levels*,* you know? People don’t understand the community healthcare worker needs the same access as a patient navigator but that’s not the same title; so that was the only thing but everybody else was like*,* “Oh yeah! I’m excited!”* –Clinic leader, HIV program grant manager

### Theme 4) funding, hiring, and retention challenges

The initiative was designed so that jurisdictions were encouraged to leverage existing funding streams as they implemented their interventions. This approach was taken to promote the *feasibility* of implementation and ultimately the long-term *sustainability* of the interventions, although key informants noted some drawbacks to this arrangement. For example, contracts to hire the CHWs were set up through the state health departments, and these positions took longer to establish than participants expected. Informants in both jurisdictions described feeling like they lost momentum with the project and noted how this delay created challenges with recruitment and onboarding because they did not know when CHWs would be available. A common theme was that organizations felt they had little control over the hiring process and integration of the CHWs. State health department leaders felt similarly frustrated because they had little authority over the funding sources used to support the new CHW positions.

One major point of disagreement was around the qualifications necessary for a CHW. An informant at the health department described the challenge of convincing their colleagues to hire someone for the CHW role.

*“We had [one potential CHW]*,* but we lost him because when he was initially interviewed*,* everybody was going*,* well*,* why would you hire him? He doesn’t have a degree*,* and he doesn’t have this*,* and he doesn’t have that. But he can relate to people. And that’s what we needed. But*,* so*,* initially we had pushback from some of the providers*,* and we had pushback from the state because the state hired them under an employment contract that they had previously and*,* you know*,* they had questions about why did you select this individual? You know? And I can only say it was – it was a gut feeling*,* but in addition to that*,* it was what we knew we needed to relate to our clients.”* – Project leader, jurisdiction-level

There were also disagreements about the scope of practice for the CHWs and where their positions should be located on a day-to-day basis, frequently due to different requirements or expectations from the funding streams that supported their positions. An informant described how they had to advocate to have the CHWs based at clinics or service agencies rather than at the local or state office, which provided funding for these positions:

*“The state had their own ideas of what the community health workers should look like…The way they did it [at another clinical site] was that the community health workers were housed in their offices -- at the state office. But for us*,* we thought it was better for them to be housed at the agency so that they understand the culture of the agency*,* the inner workings of the agency*,* the people at the agency.”* – Project leader, jurisdiction-level

The jurisdiction was able to negotiate this placement and have the CHWs based at the agencies. In both jurisdictions, ongoing challenges with clarifying CHW work-related tasks and written contracts, which were negotiated at a state level, pointed to a need to renegotiate contracts so that those would be more consistent with the CHWs’ roles in this initiative. As one health department leader noted, “we have taken ownership of the employment contract back [from the state] so that the new community health workers will be in our employ.” In this case, the amended contract gave the jurisdiction and Learning Collaborative leaders more control over crafting the job descriptions for CHWs and overseeing the hiring process across the individual clinics and agencies.

After contracts were negotiated, turnover in positions was a common challenge and raised concerns about *sustainability*. One of the programs was set up so that CHWs were shared across clinics and agencies, rather than tied to one organization exclusively. There were several instances when CHWs were so well-liked that organizations hired them separately; this arrangement then left the position unfilled at other agencies. There were other cases where CHWs went on to find jobs with higher compensation. An informant described these challenges of frequent turnover below:

*“One of the community health workers that we had hired went to the state and worked. One of the providers thought their community health worker was great and hired him on permanently under a different position and not our position. So*,* we went from five community health workers and a supervisor to two community health workers and had to rehire less than a year later.”* – Project leader, jurisdiction-level

## Discussion

This analysis reports on the experiences of leaders at state/city levels and clinic/agency representatives within their jurisdictions who integrated CHWs into their HIV care infrastructure. The stories and experiences presented in this analysis came from a larger initiative focused on the replication of evidence-informed interventions to improve HIV care-related outcomes. Our study offers insight into implementation experiences and raises important considerations as CHWs become increasingly integrated into clinical settings in the U.S. [26, 27]. Implementation support and technical assistance was provided through the Learning Collaborative model, but the interventions were not funded directly from the Learning Collaborative. Instead, jurisdictions were encouraged to use existing resources as much as possible and had some flexibility in how they implemented their programs. Overall, our findings shed light on the types of support needed to facilitate the effective integration of CHWs into clinics and agencies that provide services for individuals living with HIV. We found that although buy-in for integrating CHWs was initially mixed, informants came to value and appreciate the role of the CHW through ongoing training and guidance.

In many ways, the value of CHWs is understood and appreciated by HIV care and service sites because of their long history of working to address both medical and social needs [14]. The RWHAP provides funding for wraparound services, including case managers who help to connect patients to medical and non-medical resources. However, this context can also lead to confusion when adding CHWs to care teams due to the perceived overlap of roles. Most clinics had difficulty understanding the distinction between CHWs and case managers, and work was needed to clarify roles and responsibilities. This can be a common issue across different types of care settings but may be particularly salient in HIV care settings, where there are robust case management and peer support programs. Another study noted confusion between CHWs and case managers in HIV care settings for similar reasons [14].

Our findings and others demonstrate how work is needed to elevate the title of “Community Health Worker” [26]. This positioning is especially critical as CHWs are integrated into medical settings and may lose the more community-oriented aspect of their roles without concerted effort from the health system [16, 26, 28]. While efforts such as the Community Health Worker Core Consensus (C3) project have helped to clarify the core competencies of CHWs [2], confusion around the role of the CHW remains a common challenge [29, 30], which we saw echoed in our data. We found that role confusion can be reconciled, but it takes time and training at all levels of the health care team and across the larger system. This process was more extensive than informants had anticipated but was essential. As others have noted, efforts to delineate roles within clinical care teams can improve the work environment for CHWs and enhance the effectiveness of CHW integration [13, 31, 32]. We also observed issues around gatekeeping reflected in the narratives about role clarification, such as hesitation in granting access to the electronic health records, allowing for home visits, and defining who could be eligible for CHW positions. These examples indicated a lack of understanding about the roles of CHWs and their value. Our findings also suggested that some aspects of gatekeeping may have been related to concerns around HIV-related stigma and patient privacy, as seen in the examples of clinics expressing concerns about CHWs conducting home visits related to HIV services. Though it was not explicitly stated, these concerns may have contributed to the challenges of getting CHWs connected to the electronic health records at clinics because of increased levels of privacy protections for people living with or at risk for HIV.

Our study also highlights the challenges with fragmented funding for CHWs, an issue that has been documented extensively in the literature [33, 34]. Advocates have been calling for CHWs to be considered long-term health professionals rather than temporary solutions to address workforce shortages [33, 34], but this vision has remained challenging due to the patchwork of funding structures that support the work of CHWs [35]. Although a few states can support CHW-delivered services through Medicaid reimbursement, those typically only fund a limited range of services, and many CHW programs continue to be funded through short-term, disease-specific grants [35]. A recent change to 2024 Medicare policy will provide funding for a wider range of CHW-delivered services to address health-related social needs in clinical settings [36]. This funding stream through Medicare was not available during the time of our project, and further study will be needed to assess the implementation of this policy and its impacts on both clients and CHWs. In our study, jurisdictions were interested in developing the CHW workforce, but because the initiative did not provide direct funding for intervention delivery and there were no long-term funding mechanisms to support these roles, jurisdictions relied on CHWs who were already hired or in the process of being hired for other initiatives.

This paper adds insight into how clinics and health departments leveraged existing infrastructure to engage members of the CHW workforce, and the implications that had for intervention design and implementation. In our study, efforts to work within existing funding mechanisms made it so that individual clinics and agencies had little control over hiring and retention. We also heard stories of frequent turnover within CHW positions in the initiative, which key informants described as being connected to issues around compensation. Having sustainable funding mechanisms that support long-term CHW programs could help CHWs better integrate into health care systems and ensure that they are appropriately compensated for their work [37, 38].

In addition to funding for CHW positions, our results underscore the importance of resources directed at the clinic and institutional levels to facilitate CHW integration. The work of CHWs may not always be well recognized or understood in healthcare settings, which can cause stress and conflict among care teams, and reduce CHWs’ effectiveness in coordinating services for clients [29]. Our findings showed that additional effort was sometimes needed to undo prior negative experiences that clinicians or other staff may have had with CHW-delivered services or address narrow expectations of what the role should look like. These cases required additional coaching and technical assistance through the Learning Collaborative. Based on our findings, we recommend that before integrating CHWs into a health system, program leaders take time to understand staff assumptions about and prior experiences with CHWs. Tailored training, including opportunities for cross-training and shadowing, may be necessary to address misconceptions and promote better understanding among team members. Peer-to-peer networking also provided support to CHWs as they navigated these challenges. Networking opportunities were available through the Learning Collaboratives and other partnerships, such as local CHW associations and other professional networks. Both Louisiana and Mississippi have long histories of CHW-led workforce development and advocacy locally and nationally [39, 40], and we heard examples of how jurisdictions were able to partner with these existing efforts to support CHWs in this initiative. It is also important to underscore that having project leaders with experience as CHWs is crucial in providing responsive and effective training to clinics and advocating for CHWs. Such leaders were able to connect clinics and CHWs to other existing resources and networks, such as CHW associations, that could provide additional support and technical assistance. Furthermore, having high-level leadership engagement from CHWs is especially important in the context of clinical integration to ensure that their roles remain rooted in CHW core values and are not overly medicalized [28].

There are limitations worth noting. First, this was a small-scale exploratory analysis that was part of a larger evaluation. Our interviews focused on understanding experiences in the broader capacity building initiative, and we did not interview CHWs who were part of the care teams. Further study is needed to understand the experiences of care team members and CHWs in integrated care models over time. Although observations were conducted longitudinally, they took place at Learning Sessions, which pulled together representatives from participating clinic but did not include all team members or staff involved. In-depth interviews were only conducted at one time point, although we tried to conduct interviews midway through implementation to capture how experiences evolved over the course of the project. Further study is needed to fully explore the structural and interpersonal dynamics related to CHW integration into care teams.

Although we did not focus our interviews with people directly involved in the care teams due to the nature of our evaluation design, our interviews with health department and clinic leaders are important as these are the people who may be making decisions about hiring and training for CHWs. As more states and institutions grapple with the implications of developing and implementing sustainable funding for CHW positions, there may be more examples like this where centralized coordinating bodies (such as health departments, national organizations, payors, and advocacy groups) are responsible for overseeing CHW programs across broad networks of clinics [8]. Understanding the attitudes and perceptions that health system leaders hold of CHW programs is critical in informing further scale-up of programs. To our knowledge, this is one of the few studies that has focused on this level. Despite the limitations, this study offers valuable perspectives from health department and clinic leaders. Findings from this work can inform other state-level and large-scale efforts to integrate CHWs into health systems.

## Conclusions

This study adds to the evidence base on the value of CHWs and the resources needed to integrate them into HIV care settings. Our findings underscore the need not only for sustainable funding mechanisms for CHW positions, but also funding mechanisms that are flexible and responsive to local contexts. Funding and support must also be dedicated to clarifying roles and reinforcing the value that each member of the care team – including CHWs – can bring to ensuring high quality and comprehensive patient care.

## Electronic supplementary material

Below is the link to the electronic supplementary material.

Supplementary Material 1

## Data Availability

The interview transcripts from this study are not publicly available to protect participant confidentiality. Even with names removed from the transcripts, the details provided in the interviews could identify the participants. Summary information from the analysis can be made available from the corresponding author on reasonable request.

## Abbreviations

C3: Community Health Worker Core Consensus (C3) Project
CDC: Centers for Disease Control and Prevention
CHW: Community Health Worker
EHE: Ending the HIV Epidemic
HRSA: Health Resources and Services Administration
RWHAP: Ryan White HIV/AIDS Program
US: United States
