# Supplementary material for: Integrating community health workers into HIV care clinics: a qualitative study with health system leaders and clinicians in the Southern United States
Source: BMC Health Serv Res. 2024 Nov 1;24:1339. doi: 10.1186/s12913-024-11381-6 (PMC11531124; doi:10.1186/s12913-024-11381-6)
Supplement: Supplementary file 1 — Supplementary Material 1 [file 12913_2024_11381_MOESM1_ESM.docx]

**Baseline - Post-LS 2 Interview Guide**

*Thanks for taking part in this interview. We want to hear about your experiences with the HRSA Capacity Building Initiative in [name of jurisdiction]. Our goal is to understand how the capacity building initiative is working – what is going well, what are areas for improvement – so that we can generate recommendations for future projects. We are recording this interview, but your answers will remain confidential and your name will never be connected to your answers.*

**Introduction**

1. I’d like you to begin by describing your role within your organization.
   - How did you get into this line of work?
   - [For leadership/planning group]: What is your role in the HRSA project?
2. How did your agency/clinic/site become involved with the initiative?
   - How did you personally get involved?

**Intervention Description, Progress, and Experiences**

1. I’m curious to hear more about [intervention] – the project that you’re working on as part of the HRSA capacity building initiative. Could you tell me more about the project?
   - Probe for general project description
   - What outcomes are you hoping to see as a result of [intervention]?

*[#4. Ask of leadership]*

1. Could you tell me about how you decided on the [insert intervention] intervention/s?
   - *[Ask all:]* How well do you think the [XX] intervention model fits within your organization (or within your role in the agency)?

*[Avoid getting the Problem Statement regurgitation]*

1. What problem(s) do you hope improving, optimizing or launching the [XX] intervention will solve?
   - In what way will this work help/benefit your providers/workforce?
   - In what way will this work help/benefit clients, patients, individuals?
   - What about your organization?
2. We’re also trying to understand how this project fits into other efforts in your jurisdiction…what other similar initiatives focused on ending the HIV epidemic are taking place in the clinic/jurisdiction? What else is going on in [jurisdiction] on the EHE or other learning collaboratives?
3. At this point in time, who are the key players/staff member in terms of roles involved in the implementation process at your organization? [For leadership: who are the key players involved?]
   - Tell me more about that.
     - Probe: team structure, who decided on who to involve?
   - What kind of involvement do leaders at your organization have with the intervention(s)?
   - What kind of support have they given you/or commitment expressed? Can you give me some examples?
   - Who else would you need to get more buy-in at this point, if anyone?
     - How are you planning to get them on board, or get their buy-in if that still needs to happen?
4. [Leaders/Coordinators]: Could you tell me about the agencies and other key players that are involved in the project?
   - How did you recruit them?
   - What are they aiming to achieve through the collaborative?
   - What kinds of agencies have been more or less difficult to engage in the collaborative? How so?
5. What has been most helpful to you/your agency to prepare to launch/implement?

- How would you say the CBA/learning collaborative fits in, if at all with implementing this intervention?

1. What about challenges? What has been difficult as you implement/coordinate [intervention/LC]?
   - Probe for challenges related to LC/intervention as appropriate
2. What are the next steps with the project/intervention/learning collaborative (depending on role)?
3. Where do you foresee challenges either internal or external to your organization occurring as you get closer to/continue to implement [Intervention] in your organization?

- What do you anticipate will be most helpful in terms of facilitating implementation?
- Can you tell me about any other influences that might help or hinder effective or smooth implementation of XX intervention?
  - - - Funding*
      - Inter-organizational networks*
      - Socio-political context*
      - COVID
      - Stigma

**Learning Collaborative**

1. One of the goals for our evaluation is to understand participant experiences in the learning collaborative. By that I mean, bringing together a group of organizations to focus on making quality improvements or to implement an intervention such as [name of intervention].

- Have you participated in a learning collaborative before?
- What were your expectations when you first joined?
  - To what extent has your experience aligned with those expectations?
- What do you think about the structure/format?
  - - Probe for likes/dislikes
  - What are you/agency hoping to achieve through your involvement in the learning collaborative?

1. What would you say are the benefits of using the learning collaborative to help you [tailor for each participant, e.g., help you launch intervention at your site]? And what about the drawbacks?

- Probe: Is there anything that you feel would improve your experience in the project?

**Wrap Up**

We’re getting to the close of the interview.

1. At the end of the project (meaning when the Learning Collaborative is over), what would success look like for you?
2. Do you have any other recommendations that you would like us to pass along?
   - For CBA leadership?
   - For future capacity building projects?
   - Is there anything we haven’t talked about that you want to talk about now?
   - Who else do you recommend we interview?
   - Do we have permission to circle back with you over email to ask follow-up questions?
